# Supplementary material for: Lactobacillus paracasei HY7015 and Lycopus lucidus Turcz. Extract Promotes Human Dermal Papilla Cell Cytoprotective Effect and Hair Regrowth Rate in C57BL/6 Mice
Source: Molecules. 2022 Nov 25;27(23):8235. doi: 10.3390/molecules27238235 (PMC9738319; doi:10.3390/molecules27238235)
Supplement: Supplementary file 1 [file molecules-27-08235-s001.zip › molecules-2004803-supplementary.pdf]

Supplementary S1

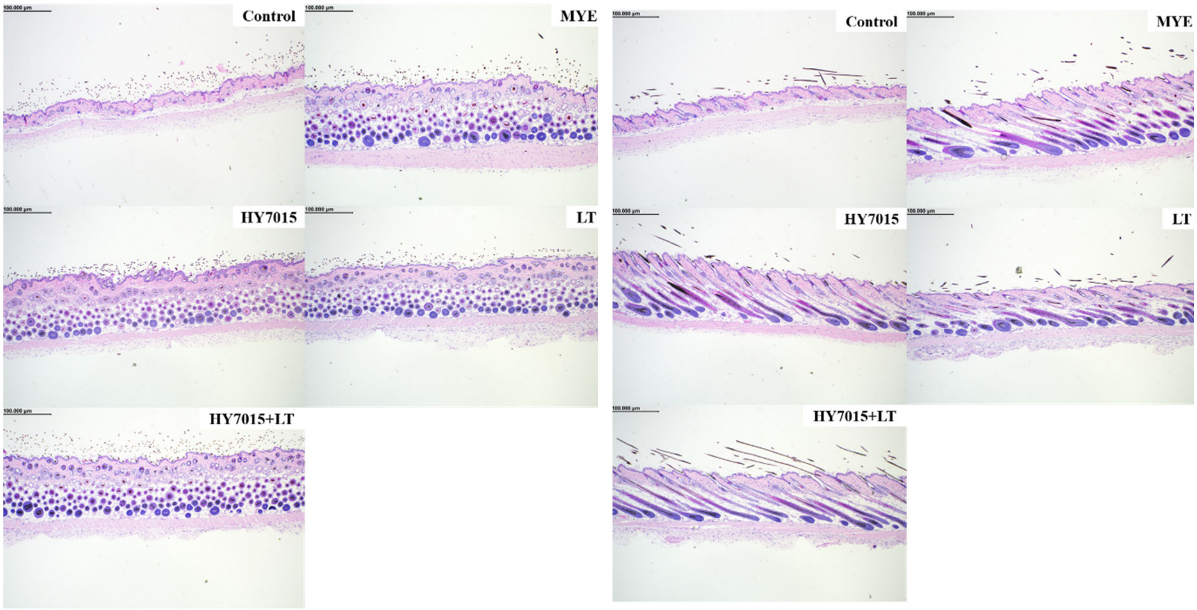

(a) **(b)**

Figure S1. (**a**) Longitudinal and (**b**) transverse sections of mouse dorsal skin tissue samples by H&E staining.

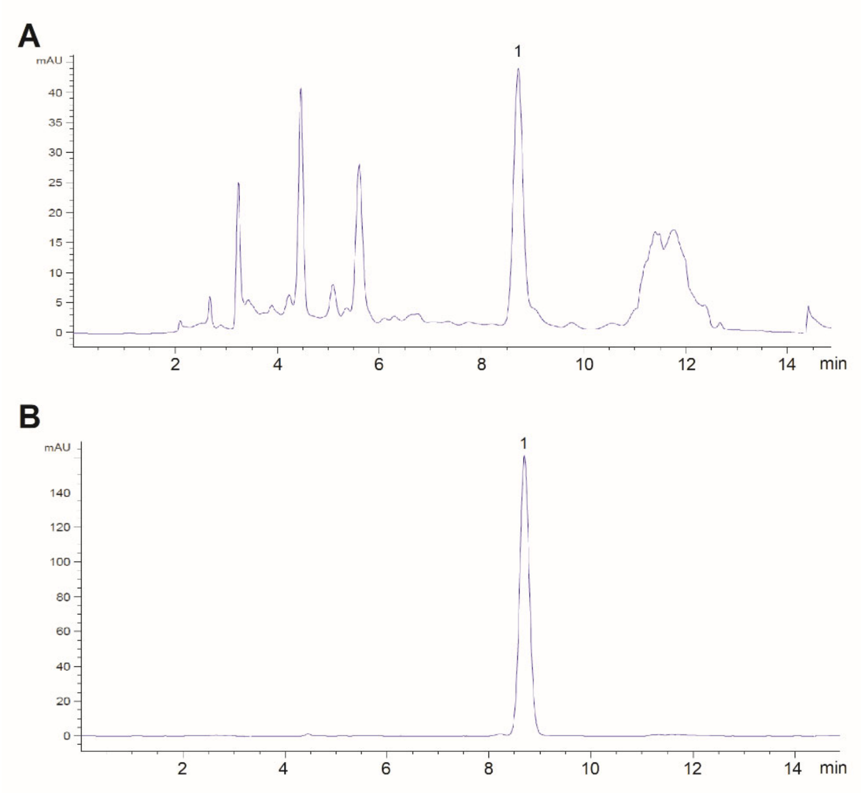

Figure S2. Liquid chromatogram of *Lycopus lucidus* Turcz. extract (**A**) and 50 μg/mL of rosmarinic acid standard (**B**). DAD detection at λ = 330 nm, 1 - rosmarinic acid.
